# Resting state default mode network is associated with wise advising

**DOI:** 10.1038/s41598-023-41408-7

**Published:** 2023-08-30

**Authors:** Chao S. Hu, Yanbin Zheng, Guang-heng Dong, Harley Glassman, Chenli Huang, Ran Xuan

**Affiliations:** 1 Department of Medical Humanities, School of Humanities, Southeast University, Nanjing, People’s Republic of China; 2 Psychological Research & Education Centre, School of Humanities, Southeast University, No. 2 Southeast University Road, Jiangning District, Nanjing, 211189 People’s Republic of China; 3 Institute of Psychological Science, Hangzhou Normal University, Hangzhou, Zhejiang Province People’s Republic of China; 4 Zhejiang Key Laboratory for Research in Assessment of Cognitive Impairments, Hangzhou, Zhejiang Province People’s Republic of China; 5 Department of Psychology, Yunnan Normal University, Kunming, Yunnan Province 650500 People’s Republic of China; 6 Ontario Institute for Studies in Education, University of Toronto, Toronto, Canada; 7 School of Psychology, University of Southampton, Southampton, UK

**Keywords:** Psychology, Human behaviour

## Abstract

Default mode network (DMN) may be associated with wisdom (i.e., mature understanding of life featured by perspectival metacognition) when advising from a self-referential perspective due to the involvement of the DMN in reflecting on personal life experiences. After a resting-state functional MRI scan, 52 adults advised some youths going through life dilemmas, half from a second-person perspective and half from a third. After advising each youth, participants indicated the psychological distance they felt between themselves and the youth. The amplitude of low-frequency fluctuation (ALFF) was measured in the DMN during resting states. Moreover, trained raters rated the participants' advice on wisdom criteria (i.e., metacognitive humility (MH), meta-level flexibility, and perspective-taking). The results showed that participants felt a significantly smaller psychological distance from the youth when advising from the second- than the third-person perspective. Moreover, only when advising from the second-person perspective was MH associated with ALFF in regions within the DMN (i.e., right rostral anterior cingulate cortex (ACC) and left dorsomedial prefrontal cortex). The right rostral ACC showed a significantly greater association with MH from the second- than the third-person perspective. Therefore, resting-state DMN activities may be important for self-involved wisdom performance (e.g., giving advice directly to others).

## Introduction

Wisdom means a mature understanding of life through learning from experience^1–4^. The ability to give advice to others (i.e., "social advising") is regarded as an important component of wisdom^1,5,6^. In the abbreviated San Diego Wisdom Scale (SD-WISE), 'social advising' is the best dimension to distinguish between the wise and the unwise^6^. In the Wise Advising Paradigm (WAP), participants provide advice to protagonists in life dilemmas (e.g., betrayal by a friend, wavering faith in morality)^7–10^. The life dilemmas used in the WAP are similar to those employed in the well-established Berlin Wisdom Paradigm^4,11^. Nevertheless, in the WAP, participants reported concrete advice to the protagonists from a second- or third-person perspective (e.g., "you/he should keep an open mind to the possibilities of the future."). In addition, the WAP uses story-based scenarios about fictional protagonists, although the stories are adapted from real-life dilemmas, and the participants were told that their advice could help these real people solve these life dilemmas.

Resting-state DMN activity may be crucial for wise advising. When one is not engaged in processing external information, the DMN is expected to undertake internally focused tasks, including self-referential processing, constructive internal reflections^12^, encoding and retrieving self-relevant stimuli^13^, flexibly simulating, anticipating and evaluating self-relevant future events, taking others' perspectives^14^, recalling personal memories, and meaning-making^15^. These processes are vital for wisdom development and performance^16,17^. Therefore, resting-state DMN activity may be associated with higher wisdom performance. Relatedly, a functional MRI (fMRI) study showed that participants with higher SD-WISE scores demonstrated more increase in the DMN activity for the contrast of moral‐personal versus moral‐impersonal dilemmas (e.g., those in which the decision-maker's choice directly violates another person's rights vs. not)^18^. Moreover, a recent electroencephalogram (EEG) study found that resting-state neural oscillations in the frontal lobe were associated with wise advising from a second- (*r* > 0.36) but not a third-person perspective^10^.

On the other hand, advising from the second-person perspective is more self-related than the third-person perspective. Previous research suggests that using the second-person pronoun "you" to refer to the self in communication is more prevalent in contexts requiring explicit self-control^19^ and performs a crucial role in introspection and meaning-making from negative personal experiences^20^. The second-person perspective implies a dynamic interaction with others, while the third-person perspective involves an impersonal, distant observation of others^21–23^. As shown by the previously mentioned EEG study, participants felt significantly less psychological distance from the protagonists when advising from a second- than a third-person perspective^10^. Psychological distance indicates the individual’s feeling that an object, person, or event is closer or farther away from the self^24^.

Consistent with the previous literature, resting-state DMN activity is expected to be associated with wise advising from the second-person perspective. To test this hypothesis, we conducted an fMRI study with the WAP to measure participants’ resting-state amplitude of low-frequency fluctuation (ALFF) before advising on various life dilemmas from second and third-person perspectives. ALFF analysis is an fMRI method related to EEG power analysis yet with greater spatial resolution. It summarizes the local brain activity’s amplitude characteristics and reliable properties in the time domain with strong temporal stability and long-term test–retest reliability that lasts roughly 6 months^25,26^.

The WAP employs life dilemmas advising that require wisdom. As one learns to adapt to the uncertainty of life, meta-level features of wisdom arise, including metacognitive humility (MH)—the awareness of one’s limitation in knowledge and intelligence, and meta-level flexibility (MF)—a preference to remain open to alternative possibilities and changes^27–29^. These meta-level features of wisdom contribute to perspective-taking (PT) when providing advice on life dilemmas, namely, imagining oneself experiencing the lives of the protagonists' life dilemmas and reasoning how they might see the world from their point of view^30^. MH, MF, and PT are generally acknowledged as reliable indicators of wisdom^27–29^.

Wisdom involves modeling the world and life challenges, a slow and effortful process^31^. Individuals are more likely to invoke wisdom for the problems they consider more important. Moreover, the self-serving bias may prevent individuals from employing wisdom. For example, when individuals are victims of interpersonal conflicts, they are less inclined to use wisdom for solving their own problems than when others are the victims^32^. Furthermore, individuals are less wise for solving problems they have less experience with or less information readily retrievable from memory^33^. Therefore, We controlled the levels of self-relevance and familiarity of the WAP tasks by using scenarios of a group of anonymous youth (See Appendix A for details).

We are the first to explore how the resting-state ALFF within the DMN relates to wise advising. Besides the DMN, we also explored how wise advising relates to the resting-state ALFF within the whole brain. Given the scarcity of literature, it is unclear how the resting-state ALFF within the whole brain relates to wise advising. Nevertheless, some theoretical speculations exist about wisdom-related brain regions outside the DMN (e.g., hypothalamus and amygdala)^34^. Besides, wise advising from the third-person perspective was not significantly correlated with the neural oscillations on the frontal lobe in the previous EEG study^10^. However, wise advising from a third-person perspective may be associated with brain regions outside the frontal lobe. Therefore, it may inform future research by exploring this issue.

To our knowledge, there is only one neurocognitive study related to wise advising, and it only investigated the resting state neural oscillation in the frontal lobe before advising^10^. Another two neurocognitive studies did not involve the resting-state. An EEG study found that score on the SD-WISE was associated with neural oscillations in the temporoparietal junction and left insula when processing happy faces^35^. The only fMRI study related to wisdom adopted a different measure for wisdom (i.e., the self-report SD-WISE) and focused on brain activities during a decision-making task^18^. Therefore, it needs to be clarified how wise advising relates to the resting-state ALFF within the brain. And we hypothesize that wise advising from the second-person perspective will be significantly positively correlated with ALFF of the frontal lobe, just as in the only neurocognitive study on wise advising^10^.

## Results

### Demographic effect

Independent-sample T-tests and correlation analyses revealed no significant effect of gender or age on any wisdom rating or psychological distance, *ps* > 0.05.

### Behavior

The wisdom performance and psychological distance for each perspective are shown in Table 1. Paired sample t-tests revealed no significant differences in any subcomponent of wisdom. However, the psychological distance was significantly smaller when advising from a second- than a third-person perspective.

**Table 1:** Wisdom scores and psychological distance when advising from different perspectives and the corresponding correlation and paired-sample t-test results.

Variable	2nd (M ± SD)	3rd (M ± SD)	r	t (51)	95% CI	*p*
Metacognitive humility	0.394 ± 0.550	0.414 ± 0.524	.32*	− 0.22	− .194, .156	.826
Meta-level flexibility	1.298 ± 0.839	1.260 ± 0.825	.61**	0.38	− .168, .244	.826
Perspective-taking	0.635 ± 0.701	0.457 ± 0.583	.42**	1.84	− .016, .372	.216
Psychological distance	2.442 ± 1.211	2.913 ± 1.115	.27	− 2.42	− .862, − .080	.038

N = 52; 2nd: second-person perspective; 3rd: third-person perspective; CI: confidence interval for the difference between the means.

*, *p* < 0.05, **, *p* < 0.01.

The *p* values are for the differences between different perspectives, adjusted by the False Discovery Rate method; *N* = 52.

### ALFF within the DMN

**Figure 1:**
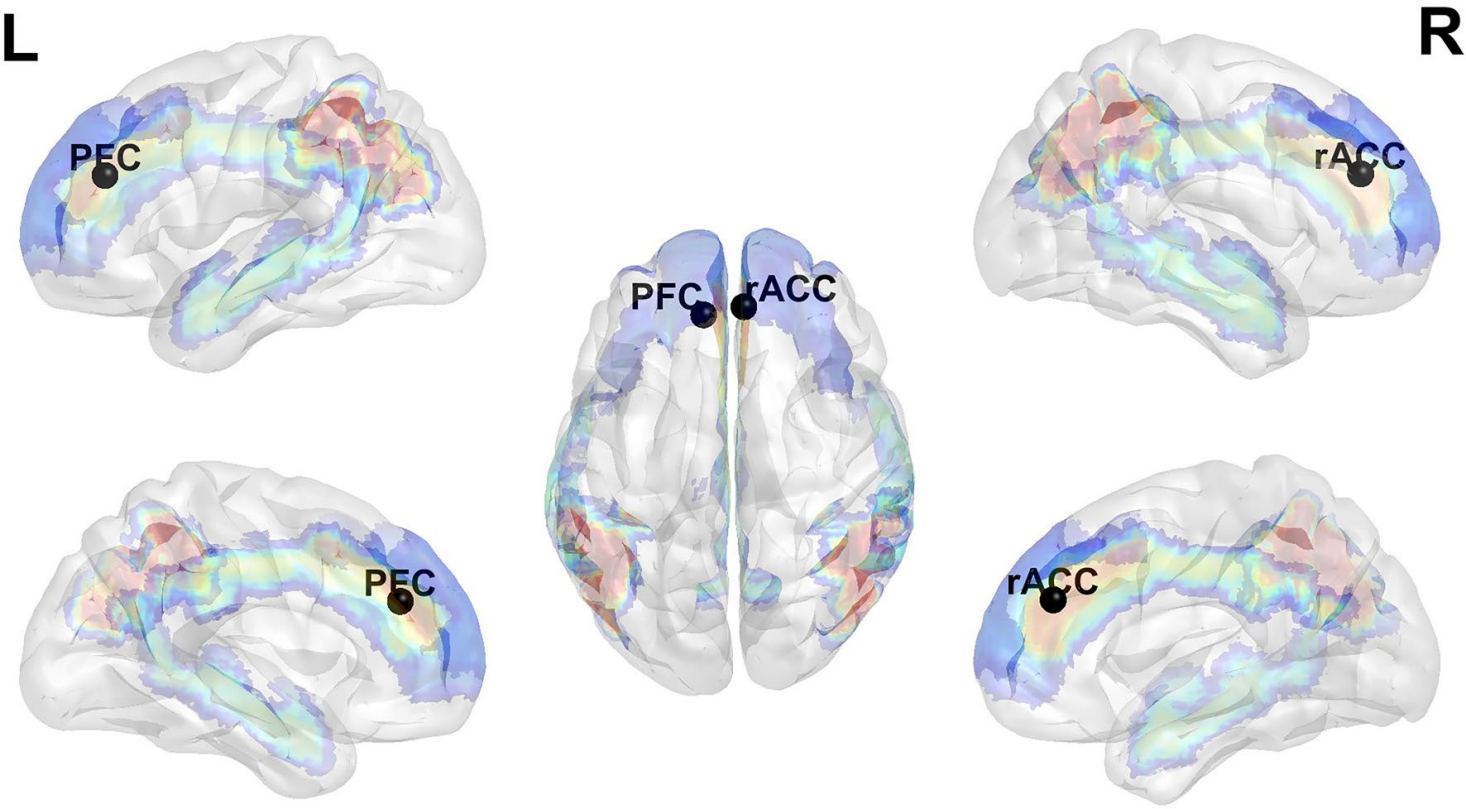
Significant correlation between resting-state ALFF within the DMN and metacognitive humility when advising from the second-person perspective. Specifically, the ocean blue mask is the regions of the dorsomedial prefrontal cortex (BA 9, 10, 24, 32), the turquoise regions located in the temporal gyrus represent the lateral temporal cortex (BA 21), the yellowish-red mask aside with node "rACC" indicates the ventral medial prefrontal cortex (BA 10, 24, 32). In the central of this image, the brain mask with light red situated under node "PFC" and node "rACC" is the inferior parietal lobule (BA 39, 40); the blue-green brain mask located between two hemispheres is the posterior cingulate cortex (BA 23, 29, 30, 31) (Note, PFC: prefrontal cortex; rACC: rostral anterior cingulate cortex).

The correlation analysis between wisdom scores and ALFF within the DMN revealed that MH was associated with ALFF in the right rostral anterior cingulate cortex (ACC) and left dorsomedial prefrontal cortex (PFC) when advising from a second-person perspective. Moreover, paired-correlation difference tests revealed that the right rostral ACC showed a significantly greater association with MH from the second- than the third-person perspective (see Table 2 and Fig. 1 for details).

**Table 2:** Significant correlations between wise advising (from 2nd or 3rd person perspective) and resting-state ALFF after Gauss Random Field correction (two-tailed).

Regions	Voxels (N)	Coordinate	Wisdom	2nd	3rd	t(49)	*p*
Left dorsomedial PFC	**15**	**− 9, 39, 27**	**MH**	.555*	**.363**	**1.39**	**.172**
Right rostral ACC	**14**	**6, 42, 27**	**MH**	.574*	**.231**	**2.46**	**.017**
Left precuneus	*52*	*− 3, − 39, 63*	*PT*	.614**	*.244*	*3.00*	*.004*
Right calcarine	*51*	*15, − 66, 18*	*PT*	.626**	*.356*	*2.22*	*.031*
Dorsal ACC	*57*	*0, 24, 27*	*MH*	*.183*	.601**	*− 3.07*	*.003*

*N*: number of the voxels; 2nd: second-person perspective; 3rd: third-person perspective; *t* and *p* values are for the differences between correlations when advising from different perspectives; *PFC* prefrontal cortex *ACC* anterior cingulate cortex.

*, *p* < 0.05, **, *p* < 0.01.

Bold font indicates results based on the correlation analyses within the DMN. *Italic font indicates results based on the correlation analyses within the whole brain*).

### ALFF within the whole brain

**Figure 2:**
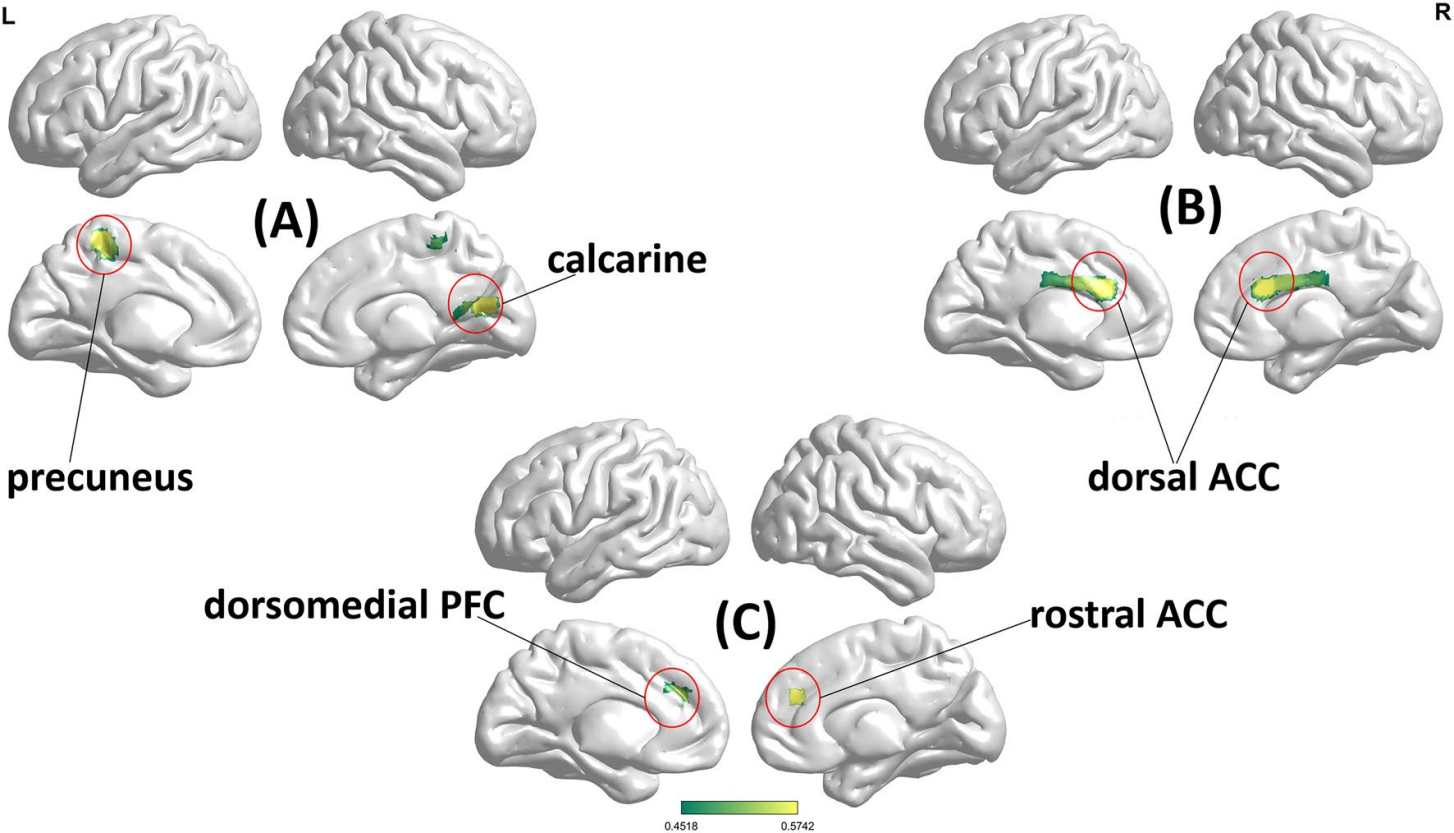
Significant correlation between resting-state ALFF within the whole brain and wisdom performance when advising from different perspectives. To be specific, (**A**) is for the significant correlation between PT of second-person perspective and resting-state ALFF; (**B**) is for the significant correlation between MH of third-person perspective and resting-state ALFF; (**C**) is for the significant correlation between MH of second-person perspective and resting-state ALFF within the DMN. MH: metacognitive humility. PT: perspective-taking. ACC: anterior cingulate cortex. PFC: prefrontal cortex (Note: circled brain regions indicate significant areas after the Gauss Random Field correction, two-tailed. See the un-thresholded map in the Supplementary Information).

The correlation analysis between wisdom scores and ALFF within the whole brain revealed that the left precuneus and right calcarine (outside the DMN) were associated with PT when advising from the second-person perspective. On the other hand, the dorsal ACC (on the border of DMN) was associated with MH from the third-person perspective. Furthermore, each of these associations was significantly stronger than its counterpart when advising from a different perspective (see Table 2 and Fig. 2 for details).

## Discussion

The present study supports our hypothesis that resting-state DMN activity is associated with wise advising from the second-person perspective. Moreover, the results revealed that wise advising from the second-person perspective was associated with distinct brain regions from the third-person perspective. Specifically, resting-state activity in the dorsomedial PFC was associated with MH from the second-person perspective (*r* = 0.555). The dorsomedial PFC is responsible for metacognition during decision-making^36,37^ and metacognitive representations of social information^38^. Our results replicate findings from the previous EEG study—demonstrating that resting-state neural oscillations in the frontal lobe are associated with MH from the second- but not the third-person perspective^10^. Additionally, one study showed that reflecting on negative personal experiences from the first-person perspective was associated with greater activity in the medial PFC—an important region of the DMN—compared to the third-person perspective^39^. Moreover, the right rostral ACC showed a significantly greater association with MH from the second- (*r* = 0.574) than the third-person perspective (*r* = 0.231). The DMN activity may be related to self-referential processing functions involved in wisdom (i.e., metacognitive humility). DMN may be related to greater self-awareness of one’s intellectual limitations, especially from a more self-related perspective (i.e., the second-person perspective).

Additionally, wise advising from each perspective is associated with resting-state activation in brain regions neighboring the DMN, such as the precuneus and calcarine. These regions were involved in directing attention to real or simulated movement in space^40^. Our results dovetail with previous findings that wisdom is associated with the cuneus and lingual gyrus^18^. Wise individuals may have robust brain systems supporting visual imagery, simulated movements between viewpoints, and greater awareness of the self and others. Furthermore, we demonstrated that the precuneus and calcarine showed a significantly greater association with PT from the second- than the third-person perspective. The resting-state activity in the precuneus and calcarine may be more related to self-related wisdom performance (e.g., imagining oneself problem-solving in social scenarios one has similarly experienced).

We found an association between MH and ACC (*rs* > 0.57). The ACC is responsible for controlling impulsivity (e.g., premeditation before decision-making)^41^, and detecting errors and conflicts^42^. The ACC may support MH by suppressing one’s impulsive judgments and reflecting on mistakes. Wisdom experts have consistently proposed that the ACC is responsible for dealing with uncertainty^34^, an essential feature of MH. Additionally, we found that the rostral ACC was related to MH when advising from the second-person perspective, while the dorsal ACC was related to MH when advising from the third-person perspective. In contrast to the dorsal ACC, the rostral ACC is believed to be associated with more self-related processes such as reward-directed decision-making^43,44^. Taken together, the dorsal and rostral ACC may have distinct functions in wisdom performance from different psychological distances.

Overall, the present study has demonstrated a relationship between resting-state brain activity and wise advising from distinct perspectives. Specifically, brain regions in the DMN involved in self-referential processing (i.e., dorsomedial PFC, rostral ACC) were associated with metacognitive humility when advising from the second-person perspective. In contrast, when advising from a third-person perspective, metacognitive humility was associated with the dorsal ACC. Similarly, the precuneus and calcarine showed a significantly greater correlation with perspective-taking from the second- than the third-person perspective. These findings may be related to the fact that the second-person perspective invites greater proximity to the self. Future studies may extend this work by exploring the involvement of the DMN in wise advising across different ages or cultures.

We live in a time when the resting-state is greatly occupied by information technology such as cell-phone. Cell-phone addiction has caused many mental problems (e.g., anxiety, stress, and depression)^45^, and the lack of a resting-state for wisdom development may be a potential mechanism. Our work provides important implications for wisdom interventions. For example, wisdom may be improved by increasing resting-state brain activity, similar to the effect of mindfulness meditation on wisdom^46^. Nevertheless, there are some limitations to this study. A man-focused dilemma scenario may lead to systematic differences in results. Future research should adopt gender/sex-neutral dilemma scenarios to see if the results are similar to this study.

## Methods

The current study was preregistered on October 24, 2019, prior to data collection (https://osf.io/jkug8/). This study was performed following the principles of the Declaration of Helsinki. The ethics committee at the Hangzhou Normal University approved all procedures used in the current study. Informed consent was obtained from all the participants after they were provided with a brief description of the study. Software G*Power 3.1 was used to perform a statistical power analysis, which indicated that a sample size of 46 with a power of 0.80 (0.05 alpha error probability rate) was required to detect the minimum effect (i.e., *r* = 0.36)—as found in the previous EEG study measuring the association between resting-state neural oscillations and wise advising^10^.

### Participants

We recruited participants from sponsoring universities through recruitment posters on campus and collected the fMRI data in a lab at the university's affiliated hospitals. We recruited 55 participants aged 18 to 26 (*M* ± *SD* = 20.80 ± 1.99; 38 males, 17 females). Two participants did not complete the advising tasks, and one showed significant artifacts from the fMRI (i.e., the head motion was more than 3 mm, while none of the other participants’ frame-wise displacement calculated by Jenkinson’s method^47^ was more than 3 mm). Thus, the final data included 52 participants (*M* ± *SD* = 20.90 ± 1.95; 36 males, 16 females). Participants were all native-born healthy Chinese participants without current or historical psychiatric, neurological, or medical conditions.

### Materials

#### Wise advising questionnaire

The questionnaire consisted of four advising tasks; two were randomly assigned to a second-person perspective, and the other two were assigned to a third-person perspective. We printed an equal number of different versions of questionnaires (e.g., "task1-2nd perspective, task2-3rd perspective, task3-3rd perspective, task4-2nd perspective", "task1-3rd perspective, task2-2nd perspective, task3-3rd perspective, task4-2nd perspective"), ensuring that each task was evenly distributed to different perspectives across participants. For each advising task, a brief description of the life dilemma of a protagonist was provided from a second- or third-person perspective. For example, a dilemma in the second-person condition includes: "I told my good friend my most important secret, but I didn't expect the friend to reveal the secret to others. For this, I am very distressed." In contrast, a dilemma in the third-person condition includes: "A young person told the best friend the most important secret, but the person did not expect this good friend would reveal the secret to others. For this, the young person was very distressed." Subsequently, participants were given the following instructions for advising from a second-person perspective: "Now please close your eyes and imagine this young person is right in front of you and needs your advice. Please try to think about how these events will develop. Why did it develop like this? What should this person do? Please refer to this person using the second-person term 'you'." Similarly, participants were instructed from a third-person perspective as follows: "Now please close your eyes and imagine this young person needing your advice. Please try to think about how these events will develop. Why did it develop like this? What should the protagonist do? Please refer to this person using the third-person term 'he'." The questionnaire states that "you are expected to provide advice to some young people going through trouble in their lives." Moreover, the scenarios are based on "troubles" in real life of Chinese youths. See the Supplementary information for a comprehensive overview of the questionnaire in English and Chinese.

### Procedure

Participants' resting-state brain activity was scanned before completing the wise advising questionnaire. Participants gave advice on some life dilemmas from the second-person perspective and some from the third (randomly allocated).

Before entering the MRI scanner, written consent was obtained from the participants. Additionally, participants completed the International Interview Examination for neuropsychiatric disorders (MINI) and Beck Depression Inventory (BDI). Participants with mental disorders, claustrophobia, metal implants, or brain injury within the past year were excluded to prevent personal injury and extraneous variability in brain activity.

All participants removed any metal item(s) from their bodies (e.g., jewelry, wallets) to avoid injury and interference from the fMRI. Once participants entered the scanner, they were instructed to keep their eyes closed and lay motionless to reduce head motion during scanning. A sponge filling was used to fill the space outside the head to reduce movement and the impact of noise on participants.

#### Scanning

Twenty participants were scanned with a 3-Tesla Siemens Trio scanner using a gradient-echo EPI sequence in 33 axial slices with echo time (TE) = 30 ms, repetitive time (TR) = 2000 ms, flip angle = 90°, slice thickness = 3 mm, field of view (FOV) = 220 × 220 mm^2^, matrix size = 64 × 64, and resting-state images consisting of 240 functional volumes. Another 32 participants were scanned on a 3-Tesla GE MR 750 scanner with a gradient-echo EPI sequence in 43 axial slices with TE = 30 ms, TR = 2000 ms, flip angle = 90°, slice thickness = 3.2 mm, FOV = 220 × 220 mm^2^, matrix size = 64 × 64, and resting-state images consisting of 240 functional volumes. There was no significant difference in wisdom ratings or ALFF values between the participants who entered different scanners (all *p* > 0.05).

#### Advising

After receiving an eight-minute fMRI scan, participants randomly drew a questionnaire and completed the advising tasks on a pen-and-paper questionnaire. After advising each protagonist, the participants indicated the psychological distance they felt between themselves and the protagonist on a 7-point Likert Scale (0—no distance, 6—furthest distance).

### Data analysis

#### Rating of wise advising

Following the procedures of previous studies on WAP^7–10^, two raters naive to the purpose of the experiment were trained on sample advising transcripts until they reached high inter-rater reliability. These trained raters then rated each of the participants' advice on the four life dilemmas with each of the wisdom subcomponents: metacognitive humility (MH), meta-level flexibility (MF), and perspective-taking (PT).

The raters rated the transcripts on a 5-point scale (0 = no instance fitting the criterion, 4 = entirely fitting the criterion). Inter-rater reliability of wisdom ratings for advising on each life dilemma (i.e., D1–D4) on each subcomponent (i.e., MH, MF, PT) reached a statistically acceptable level of agreement. The average measure of Intra-class Correlation Coefficients (ICC) with a two-way random effects model are as follows: 0.74 (D1, MH), 0.75 (D2, MH), 0.79 (D3, MH), 0.80 (D4, MH); 0.80 (D1, MF), 0.74 (D2, MF), 0.84 (D3, MF), 0.63 (D4, MF); 0.92 (D1, PT), 0.67 (D2, PT), 0.67 (D3, PT), 0.76 (D4, PT). Ratings from all raters were averaged to get the final scores of MH, MF, and PT for advising on each life dilemma. Finally, the average scores of MH, MF, and PT when advising were calculated for the second- and third-person perspective, respectively.

#### Analyses of fMRI data

Functional images were preprocessed with the DPARSF toolbox in MATLAB 2014b^48^. Initially, we translated data formats from DICOM to NIFTI and discarded the first ten time-point slices to improve the data quality. Next, slice-timing corrections were conducted with the middle slice as the reference slice. Then, the data was reoriented and realigned to the first slice. Participants with maximum displacement in any direction larger than 3.0 mm or head motion bigger than 3.0 mm were excluded. We used realign function of DPABI combined with the algorithm of FD-Jenkinson to calculate the participants' head motion data with XYZ translation and rotation^49^. After we excluded undesirable data (i.e., translation more than 3 mm, rotation more than 3 degrees), we used realign function to correct the head motion. Moreover, we checked the head motion (i.e., lower than 3 mm) and data quality (i.e., no artifacts) for each participant to ensure the quality of images was acceptable for analyses. Subsequently, the data were spatially normalized to the standard Montreal Neurological Institute (MNI) space using EPI (i.e., echo planar imaging) template and re-sliced to 3 × 3 × 3 mm voxels. Besides, the ALFF of each voxel is divided by the average ALFF of all voxels in the whole brain to obtain the mALFF for each voxel. Subsequently, the data were smoothed using a 4-mm full width at half maximum (FWHM) isotropic Gaussian kernel. Additionally, several sources of spurious variances were removed by the regression algorithm, including the head motion parameters, linear drift, global BOLD signals, and BOLD signals in white matter and cerebrospinal fluid. Finally, we calculated the resting-state ALFF following the procedure in previous studies^25^. Specifically, the time series of any given voxels was transformed through the Fast Fourier Transform (FFT), then the ALFF was calculated as the average square root of power in the low-frequency band (0.01–0.1 Hz).

In the DPABI software, we combined the AAL template with the BA area and then resliced the mask to define DMN. Previous studies indicate that the ventral medial prefrontal cortex (BA 10, 24, 32), posterior cingulate cortex (BA 23, 29, 30, 31), inferior parietal lobule (BA 39, 40), lateral temporal cortex (BA 21), and dorsomedial prefrontal cortex (BA 9, 10, 24, 32) are essential regions for a DMN mask^12,14^. Thus, we viewed the AAL template mask in the Viewer function and optioned these regions in DAPBI. Then we constructed these regions as the grey mask and resliced it into a 3 × 3 × 3 mask.

We calculated the whole brain ALFF first, then used a DMN mask to extract the DMN-ALFF value and get the ALFF within the DMN. For the whole-brain analysis, the whole-brain grey matter mask was applied. After getting the whole-brain grey matter, we checked the mask quality visibly using MRIcro software. Then we renormalized and resliced the mask to guarantee the mask could be used in later analyses. Subsequently, a correlation analysis between ALFF within the DMN and wisdom scores (i.e., MF, MH, PT) was conducted in the DAPAI, with gender, age, and scanner as covariates. Additionally, the Gauss Random Field (GRF) correction (two-tailed) was used for multiple corrections. Specifically, we selected the voxels with *p* < 0.001 and the clusters with *p* < 0.05. Similarly, the relationship between ALFF within the whole brain and wisdom scores (i.e., MF, MH, PT) was investigated with a correlation analysis (with GRF correction, two-tailed). Finally, for each significant correlation between the ALFF and wisdom score, we analyzed its difference with the corresponding correlation when advising from the other perspective.

### Supplementary Information

Supplementary Information 1.

Supplementary Information 2.

## Data Availability

All data are available at: https://osf.io/dhpey/?view_only=388920cd5052489fb3a73ef4a63bf015.
