# Supplementary material for: Resting state default mode network is associated with wise advising
Source: Sci Rep. 2023 Aug 30;13:14239. doi: 10.1038/s41598-023-41408-7 (PMC10468530; doi:10.1038/s41598-023-41408-7)
Supplement: Supplementary file 1 — Supplementary Information 1. [file 41598_2023_41408_MOESM1_ESM.docx]

**Appendix A**

**An example of the Questionnaires of Wise advising used (English version)**

Number: ______ Gender: _________ Age: _____

Hello, thank you for participating in our research. Next, you are expected to provide advice to some young people going through trouble in their lives! Some people may prefer your suggestions from the third-person perspective, while others prefer the second-person perspective. Therefore, for two questions, please use the second-person perspective to give suggestions (for example: "what should you think, what should you do"); for the other two questions, please use the third-person perspective to give suggestions (for example: "what should he/she think, what should he do").

You will see four questions and corresponding requirements. Please read the questions carefully and answer them as required. There is no right or wrong answer. Your hard work and seriousness will play a key role in our research! It may even change the lives of many people! Here, thank you in advance for your contribution!

Question 1. The second-person perspective

A young person said: "I told my best friend the most important secret. I didn't expect this best friend to leak the secret to others. For this, I was very distressed."

Now please close your eyes and imagine: this young person is right in front of you and wants to hear your thoughts and suggestions. Would you please think about how things will develop? Why did it develop like this? What should the person do? Please call this young person in the second person "you" and write down your thoughts and suggestions that you would like to tell the young person in front of you in the box below.

Suggestion: I think you

Excuse me, when you provided the advice just now, how much distance did you feel to this young person: 0-no distance, 1-very close, 2-relatively close 3-moderate, 4-relatively far, 5-very far, 6-infinitely distant. Please choose a number that reflects your true feelings.

Question 2. The third-person perspective

A young person thinks that a kind person is often not rewarded, but a person who pretends to be kind is more likely to succeed, so he/she doubts whether to be a kind person.

Now please close your eyes and imagine this young person needs your advice and wants to hear your thoughts and suggestions. Would you please think about how things will develop? Why did it develop like this? What should the person do? Please address this young person in the third person "he" and write down your thoughts and suggestions that you would like us to convey to him/her in the box below.

Suggestion: I think he

Excuse me, when you provided the advice just now, how much distance did you feel to this young person: 0-no distance, 1-very close, 2-relatively close 3-moderate, 4-relatively far, 5-very far, 6-infinitely distant. Please choose a number that reflects your true feelings.

Question 3. The third-person perspective

There is a young person who thinks that many people can succeed without going to university, and many university students cannot find a suitable job after graduation, so this young person doubts whether going to university is genuinely meaningful. He felt very confused.

Now please close your eyes and imagine this young person needs your advice and wants to hear your thoughts and suggestions. Would you please think about how things will develop? Why did it develop like this? What should the person do? Please address this young person in the third person "he" and write down your thoughts and suggestions that you would like us to convey to him/her in the box below.

Suggestion: I think he

Excuse me, when you provided the advice just now, how much distance did you feel to this young person: 0-no distance, 1-very close, 2-relatively close 3-moderate, 4-relatively far, 5-very far, 6-infinitely distant. Please choose a number that reflects your true feelings.

Question 4. The second-person perspective

A young person said: "I am bankrupt and owe a debt that I cannot pay off for a lifetime. My relatives and friends despise me. I want to end my life."

Now please close your eyes and imagine this young person is right in front of you and wants to hear your thoughts and suggestions. Would you please think about how things will develop? Why did it develop like this? What should the person do? Please call this young person in the second person "you" and write down your thoughts and suggestions that you would like to tell the young person in front of you in the box below.

Suggestion: I think you

Excuse me, when you provided the advice just now, how much distance did you feel to this young person: 0-no distance, 1-very close, 2-relatively close 3-moderate, 4-relatively far, 5-very far, 6-infinitely distant. Please choose a number that reflects your true feelings.

**Appendix. An example of the Questionnaires of Wise advising used (English version)**

编号： ; 性别： ; 年龄：

您好，谢谢您参加我们的研究。接下来，我们希望您给一些人生困境中的青年一些建议！有些人可能更喜欢您从第3人称视角建议，而有些人更喜欢第2人称视角。所以，其中有2道题目请您用第2人称视角给建议（例如："你应该怎样想、你要怎样做"）；另外2道题目请您用第3人称视角给建议（例如："他应该怎样想、他要怎样做"）。

您将看到4个问题和相应的要求，请务必仔细阅读问题并按照要求进行回答，回答没有对错之分。您的努力和认真将会对我们的研究起关键性的作用！甚至可能改变许多人的命运！在此，预先感谢您的认真回答！

问题一. 第2人称视角

一个青年说："我把最重要的秘密告诉了自己的好朋友，没想到这个好朋友又把秘密泄露给了别人。为此，我很苦恼。"

现在请您闭眼想象一下：这个青年就在您面前，想听听您的想法和建议。请您思考事情会怎样发展？为什么会这样发展？当事人应该怎么做？请以第二人称"你"来称呼这个青年，在下列框中写下您希望告诉眼前这青年的想法和建议。

建议：我觉得你

请问，您刚才建言时，在心里觉得与这个青年的距离：0-没有距离，1-很近，2-比较近3-适中，4-比较远，5-很远，6-无限遥远。（请选择一个数字反映您心里真实的感受）

问题二. 第3人称视角

有个青年觉得善良的人往往得不到好报，而伪装善良的人却更容易成功，因此他怀疑是否要做一个善良的人。

现在请您闭眼想象一下：这个青年正需要您的忠告，想听听您的想法和建议。请您思考事情会怎样发展？为什么这样发展？当事人应该怎么做？请以第三人称"他"来称呼这个青年，在下列框中写下您希望我们转告他的想法和建议。

建议：我觉得他

请问，您刚才建言时，在心里觉得与这个青年的距离：0-没有距离，1-很近，2-比较近3-适中，4-比较远，5-很远，6-无限遥远。（请选择一个数字反映您心里真实的感受）

问题三. 第3人称视角

有个青年认为，很多人没有上大学也能成功，而许多大学生毕业后却找不到合适的工作，因此这个青年很怀疑上大学是否真的有意义。他感到很迷茫。

现在请您闭眼想象一下：这个青年正需要您的忠告，想听听您的想法和建议。请您思考事情会怎样发展？为什么这样发展？当事人应该怎么做？请以第三人称"他"来称呼这个青年，在下列框中写下您希望我们转告他的想法和建议。

建议：我觉得他

请问，您刚才建言时，在心里觉得与这个青年的距离：0-没有距离，1-很近，2-比较近3-适中，4-比较远，5-很远，6-无限遥远。（请选择一个数字反映您心里真实的感受）

问题四. 第2人称视角

一个青年说："我破产了，欠了一辈子也还不清的债，亲戚朋友都瞧不起我。我想结束自己的生命。"

现在请您闭眼想象一下：这个青年就在您面前，想听听您的想法和建议。请您思考事情会怎样发展？为什么会这样发展？当事人应该怎么做？请以第二人称"你"来称呼这个青年，在下列框中写下您希望告诉眼前这青年的想法和建议。

建议：我觉得你

请问，您刚才建言时，在心里觉得与这个青年的距离：0-没有距离，1-很近，2-比较近3-适中，4-比较远，5-很远，6-无限遥远。（请选择一个数字反映您心里真实的感受）
