# Supplementary figures and images for: Resting state default mode network is associated with wise advising
Source: Sci Rep. 2023 Aug 30;13:14239. doi: 10.1038/s41598-023-41408-7 (PMC10468530; doi:10.1038/s41598-023-41408-7)

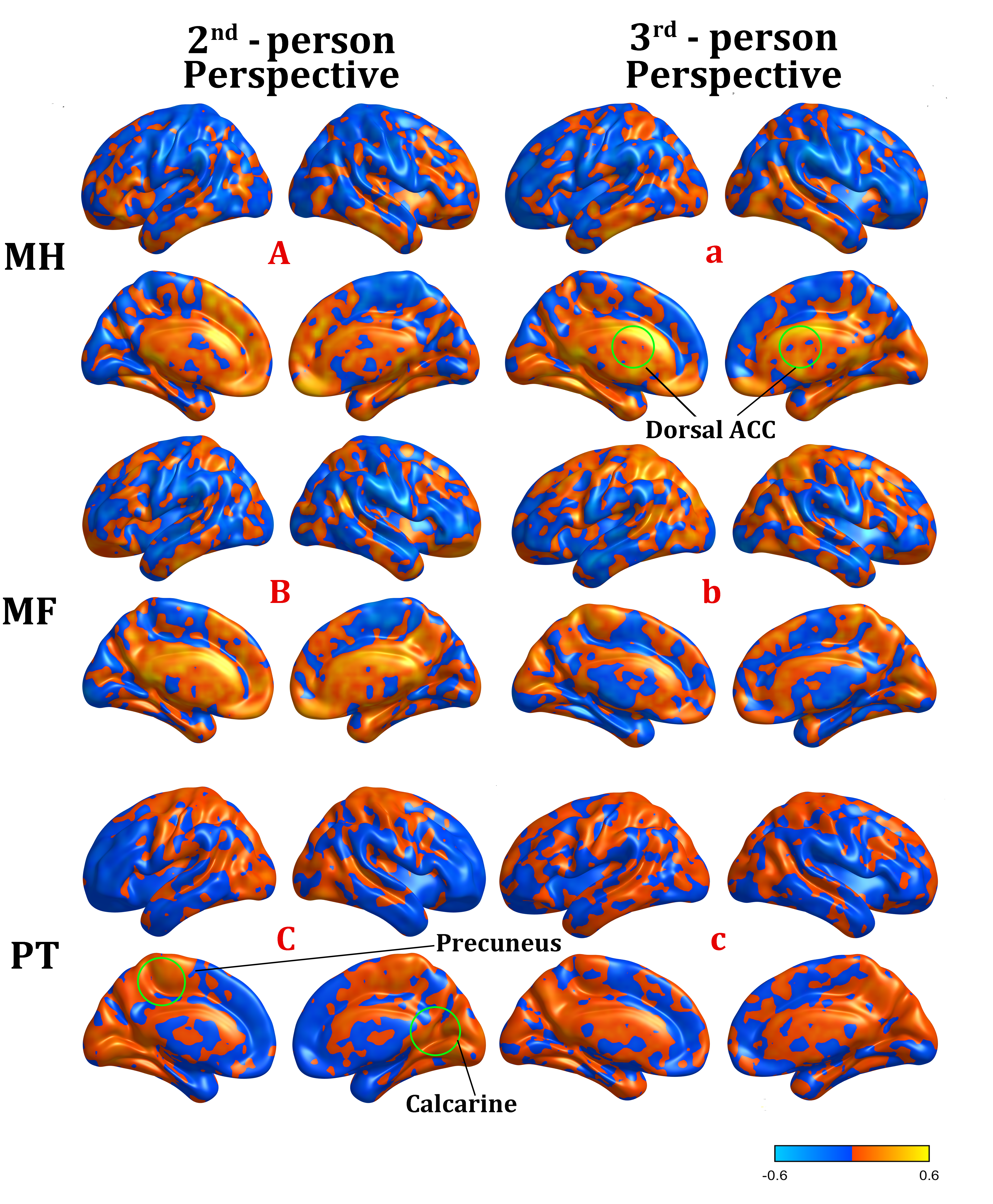

Supplement: Supplementary file 2 — Supplementary Information 2. [file 41598_2023_41408_MOESM2_ESM.png]
